# Microstructures and Enhanced Mechanical Properties of (Zr, Ti)(C, N)-Based Nanocomposites Fabricated by Reactive Hot-Pressing at Low Temperature

**DOI:** 10.3390/ma16062145

**Published:** 2023-03-07

**Authors:** Mengmeng Zhang, Boxin Wei, Lanqing Liang, Wenbin Fang, Lei Chen, Yujin Wang

**Affiliations:** 1 School of Materials Science and Chemical Engineering, Harbin University of Science and Technology, Harbin 150040, China; 2 Heilongjiang Provincial Key Laboratory of Light Metal Materials Modification and Green Forming Technology, Harbin University of Science and Technology, Harbin 150040, China; 3 Key Laboratory of Advanced Structural-Functional Integration Materials & Green Manufacturing Technology, Harbin Institute of Technology, Harbin 150001, China

**Keywords:** (Zr, Ti)(C, N)-based composites, reactive hot-pressing, solid solution, mechanical properties

## Abstract

Dense and enhanced mechanical properties (Zr, Ti)(C, N)-based composites were fabricated using ZrC, TiC_0.5_N_0.5_, and Si powders as the raw powders by reactive hot-pressing at 1500–1700 °C. At the low sintering temperature, both (Zr, Ti)(C, N) and (Ti, Zr)(C, N) solid solutions were formed in the composites by adjusting the ratio of ZrC to TiC_0.5_N_0.5_. During the sintering process, the Si added at a rate of 5 mol% reacted with ZrC and TiC_0.5_N_0.5_ to generate SiC. With the increase in Si addition, it was found that the residual *β*-ZrSi was formed, which greatly reduced the flexural strength of composites but improved their toughness. The reaction and solid-solution-driven inter-diffusion processes enhanced mass transfer and promote densification. The solid solution strengthening and grain refinement improved the mechanical properties. The ZrC–47.5 mol% TiC_0.5_N_0.5_–5 mol% Si (raw powder) composite possessed excellent comprehensive performance. Its flexural strength, Vickers hardness, and fracture toughness were 508 ± 33 MPa, 24.5 ± 0.7 GPa, and 3.8 ± 0.1 MPa·m^1/2^, respectively. These reached or exceeded the performance of most (Zr, Ti)(C, N) ceramics reported in previous studies. The lattice distortion, abundant grain boundaries, and fine-grained microstructure may make it possible for the material to be resistant to radiation.

## 1. Introduction

Zirconium carbide (ZrC) exhibits uniquely comprehensive properties such as a high melting point (~3540 °C), high hardness (~20 GPa), excellent abrasion and corrosion resistance, good thermal/electrical conductivities, and good irradiation damage tolerance [1,2,3,4]. Combining these characteristics makes ZrC a promising candidate that can be applied to next-generation rocket engines as structural components [5,6]. ZrC is also considered a potential material in the framework of the generation-IV nuclear energy system due to its excellent resistance to corrosion by fission products and low neutron absorption cross-section [7,8,9]. Introducing lattice defects, such as abundant grain boundaries and lattice distortions, can further enhance the radiation resistance of ceramics [10].

However, the densification temperature of ZrC is very high due to its strong covalent bond and low self-diffusion coefficient, which limits its application. Adding transition metal carbide, nitride, and carbonitride such as TiC, TiN, and TiCN to the ZrC matrix to form solid solutions can improve sintering performance [11,12,13]. The formation of a solid solution can promote densification, and the lattice distortion caused by a solid solution can produce a solid solution strengthening effect [14,15]. Since ZrC and TiC have the same crystal structure and a similar covalent radius, they can form continuous solid solutions [16]. At a TiC composition of under 20 mol%, however, the densification temperature of ZrC-TiC ceramics is as high as 2100 °C [17]. For ZrC-TiN and ZrC-TiCN ceramics, the formation of a (Zr, Ti)(C, N) solid solution causes more lattice distortion and improves the mechanical properties of materials. However, the stability of N in TiCN is better compared to that in TiN at high temperatures and in a vacuum [18]. Therefore, TiCN is not easily denitrified to form N_2_ during sintering, which is conducive to the densification of ceramic.

Nevertheless, ZrC-TiCN ceramics have poor densification and mechanical properties due to the high sintering temperatures. Reactive hot-pressing is considered an effective method of preparing ceramic composites. Reactions during sintering can significantly enhance the low-temperature densification behavior compared with non-reactive processes [19,20]. For ZrC and TiC, silicon (Si) is commonly chosen as a reactive sintering aid. The reaction of carbide with Si can evidently promote densification [21,22], and the product SiC can improve mechanical properties by inhibiting grain growth [23,24]. Furthermore, nanoparticle-reinforced composites are an important method to improve material properties [25,26,27].

In this study, dense (Zr, Ti)(C, N)-based composites were prepared by reactive hot-pressing at 1500–1700 °C. Si was chosen as a sintering aid to reduce the sintering temperature and enhance densification by using Si to generate the liquid phase during the sintering process. At the low sintering temperature, thermodynamic calculation showed that the molar ratio of ZrC to TiC_0.5_N_0.5_ should be 50:50 to obtain composites with Zr-rich (Zr, Ti)(C, N) and Ti-rich (Ti, Zr)(C, N) solid solutions that improve mechanical properties. ZrC-TiC_0.5_N_0.5_ (molar ratio 50:50) with 0, 5, 10, and 20 mol% Si additions were chosen as the designed compositions. The effects of Si addition on the microstructure and mechanical properties were investigated. This research will propose a new alternative for designing and preparing nuclear materials.

## 2. Materials and Methods

Commercial ZrC (*d*_av_ = 0.25 μm, purity > 99.7 wt%), TiC_0.5_N_0.5_ (*d*_av_ = 0.23 μm, purity > 99.4 wt%), and Si (*d*_av_ = 1.29 μm, purity > 99.9 wt%) were chosen as the initial powders. The powder mixtures with different compositions were milled with a ball-to-powder ratio of 30:1 in weight under an argon atmosphere. The milling time was 24 h, and the rotating speed was 250 rpm. Then, the powders were passed through an 80-mesh sieve. Table 1 shows the designed material compositions, sintering parameters, and codes.

**Figure 1:**
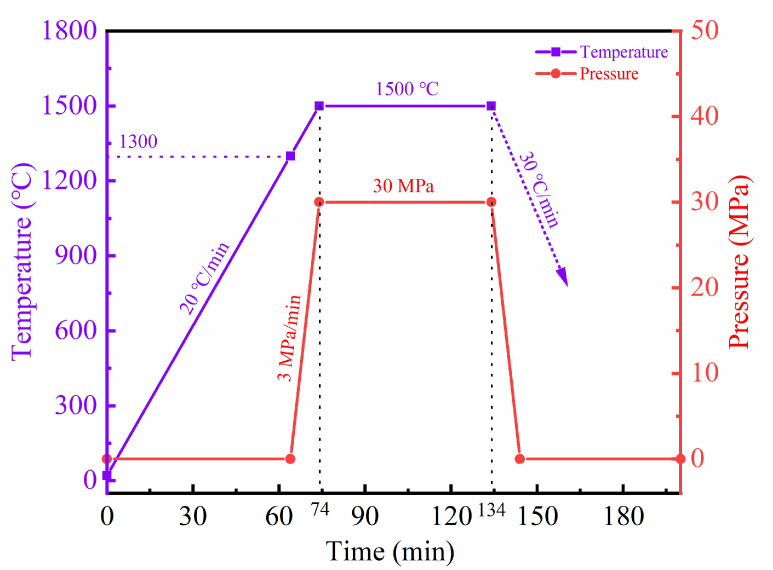
Sintering curve of Z50T10S.

Hot pressing was performed in a vacuum hot-pressing sintering furnace (AVS, model 1540, Ayer, MA, USA) using a 20 mm inner diameter high-density graphite die. The room temperature was 20 °C, and the sintering curve of Z50T10S is shown in Figure 1. The other samples’ sintering parameters are the same as those of Z50T10S, except for the sintering temperatures. The powder mixture dwelled in a vacuum for 1 h at the designed sintering temperature and pressure. The load on the mold was then removed, and the furnace cooled at a speed of 30 °C/min.

The entire surface of the samples was ground to remove the graphite foil residual and the reaction layer before being polished with diamond paste. An X-ray diffractometer (Empyrean, PANalytical Corp., Almelo, The Netherlands) was used for identifying the phase constituents. The lattice parameters were determined using Si powder as the internal standard in the 2θ range of 20°–90°. The bulk densities of the specimens were measured by Archimedes’ principle. Microstructures were observed by a scanning electron microscope (SEM, Hitachi SU5000, Tokyo, Japan) equipped with energy-dispersive spectroscopy (EDS, AZTEC, Oxford Instrument, High Wycombe, UK) for elemental analysis. The grain size and content of each phase in the specimen were obtained by image analysis using the Image-Pro Plus software (version 7.0). Transmission electron microscopy (TEM, FEI Talos F200X, Waltham, MA, USA) was used for a more detailed analysis of the microstructure.

The flexural strength was performed utilizing three-point bending (Instron-1186) with a 14 mm span and a crosshead speed of 0.5 mm/min (2 × 4 × 16 mm^3^ test bars). The reported strength values were the average tested values on five bars where the surfaces were polished and chamfered. Vickers hardness (*H_v_*) was measured using the Vickers indentation method (HVS-30) with a load of 9.8 N for 10 s. Fracture toughness (*K_IC_*) was evaluated from the as-indented crack lengths by the Evans [28] formula with the indentation method. The average *H_v_* and *K_IC_* were obtained from at least 10 indentations. The phase diagram based on thermodynamics was calculated by the software FactSage (Version 7.1) using databases from SpMCBN 7.0.

## 3. Results and Discussion

### 3.1. Phase Constitutions

**Figure 2:**
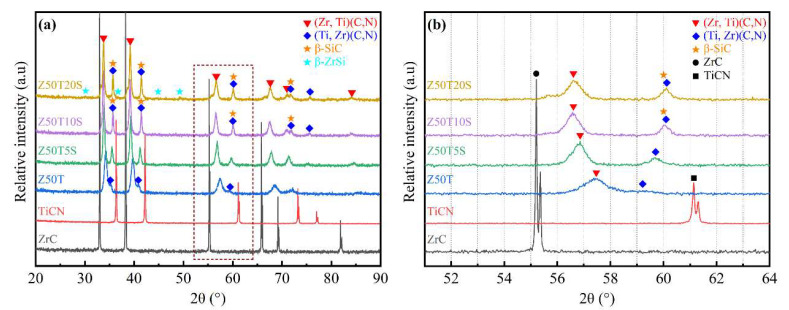
(**a**) XRD patterns of bulk ZrC, TiCN, and composites; (**b**) the expanded patterns of (**a**).

Figure 2 presents the XRD patterns of the (Zr, Ti)(C, N)-based composites. In Figure 2a, the prepared ceramics containing Si were mainly composed of two solid solution phases, including (Zr, Ti)(C, N) and (Ti, Zr)(C, N). For the Z50T sample, due to the high sintering temperature (2000 °C), a single-phase solid solution tended to form [12], resulting in the intensity of the diffraction peak corresponding to (Ti, Zr)(C, N) being very weak. On the contrary, the intensity of the (Ti, Zr)(C, N) diffraction peaks of the other ceramics containing Si was significantly enhanced but was far less than that of (Zr, Ti)(C, N). It was revealed that the formation of the (Zr, Ti)(C, N) solid solution predominated at both low and high temperatures. A similar situation also occurs in the ZrC-TiC and ZrC-TiC_0.5_N_0.5_ systems [12,29]. The phenomenon might be related to the difference between the diffusion activation energy of the Ti atom in the ZrC and that of the Zr atom in the TiC_0.5_N_0.5_ at the same temperature. However, further investigation should be carried out to verify this. With the increase in Si addition, the *β*-ZrSi was detected in the Z50T20S sample. The expanded XRD patterns are shown in Figure 2b. The (220) diffraction peaks of (Zr, Ti)(C, N) composites shifted to a region of higher angles than monolithic ZrC. In contrast, all (220) diffraction peaks of (Ti, Zr)(C, N) moved to a region of lower angles compared to monolithic TiC_0.5_N_0.5_. However, these (220) diffraction peaks mentioned above became progressively less shifted with the decreasing sintering temperature. The full width at half maximum of the (Zr, Ti)(C, N) and (Ti, Zr)(C, N) solid solutions (220) crystal plane diffraction peaks gradually decreases with the addition of Si. According to Scherrer’s formula, it can be assumed that the grain size of both solid solutions grows with the Si addition.

The diffraction peaks’ shift indicated that the lattice parameters of their corresponding phases also changed. The lattice parameters of composites were calculated by the XRD patterns (Table 2). The lattice parameter of (Zr, Ti)(C, N) decreased from 0.4696 nm (ZrC) to the minimum value of 0.4525 nm (Z50T), while the lattice parameter of (Ti, Zr)(C, N) increased from 0.4275 nm (TiCN) to the maximum value of 0.4418 nm (Z50T). This is because the (Zr, Ti)(C, N) solid solution is formed by the dissolution of Ti and N atoms with small atomic radii into the ZrC lattice; the (Ti, Zr)(C, N) solid solution is formed by dissolving the larger atomic radius Zr atoms into the TiC_0.5_N_0.5_ lattice. Compared with the lattice parameters in the Z50T sample, those of the (Zr, Ti)(C, N) and (Ti, Zr)(C, N) rose from 0.4525 nm to 0.4589 nm and decreased from 0.4418 nm to 0.4346 nm in the Z50T20S, respectively. This is attributed to the solid solubility decreasing with the reduction in sintering temperature, which also made the offset of the corresponding (220) diffraction peaks smaller, as shown in Table 2.

*β*-ZrSi (CrB-type structure) exists above 1460 °C, and transitions to *α*-ZrSi (FeB-type structure) occur below this temperature according to the Si-Zr phase diagram [30]. Nevertheless, *β*-ZrSi was found in the Z50T20S sample at room temperature, and its (002) diffraction peak shifted from 48.5 to 49.5 compared to the PDF card (# 04-004-7165), as shown in Table 2. The above phenomenon, combined with our previous study [10], indicates that Ti atoms dissolved into the *β*-ZrSi lattice to form a (Zr, Ti)Si solid solution (CrB-type) and inhibited the transition from the *β* to the α.

**Figure 3:**
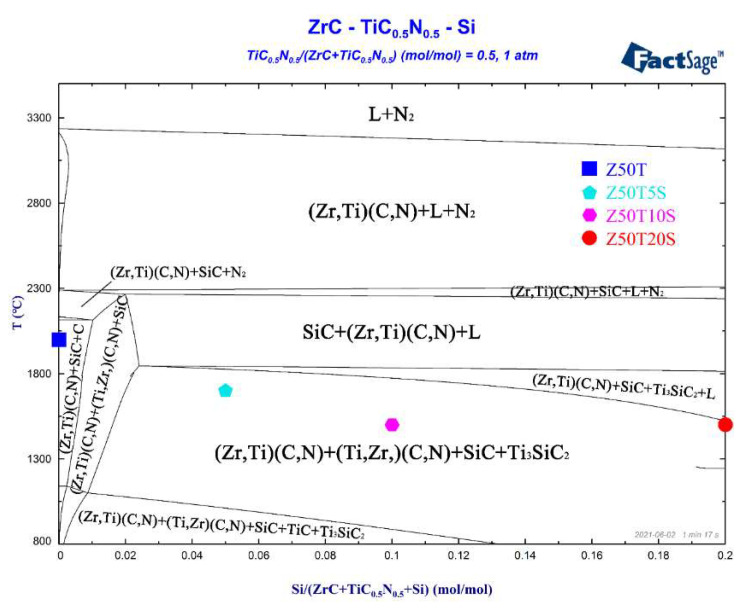
Equilibrium phase diagram calculated by FactSage of ZrC-TiC_0.5_N_0.5_-Si.

To further investigate the reaction processes of ZrC, TiC_0.5_N_0.5_, and Si during sintering, the equilibrium phase diagram was calculated using FactSage software (Figure 3). Samples with different Si additions fell within a “(Zr, Ti)(C, N) + (Ti, Zr)(C, N) + SiC + Ti_3_SiC_2_ region.” The thermodynamic calculation could reflect the reaction trend in the ZrC-TiC_0.5_N_0.5_-Si system to some extent. It was reported that ZrC can react with Si to form ZrSi below 1300 °C, and then the carbon atoms in ZrC could react with the ZrSi to form SiC and ZrC_1−x_ [22]. Therefore, there may be two stages between the raw powders during the sintering process. The first stage of the reaction occurs below 1300 °C, as follows:

ZrC (s) + Si (s) → SiC (s) + α-ZrSi (s) (1)

Simultaneously, interdiffusion between ZrC and TiC_0.5_N_0.5_ forms (Zr, Ti)(C, N) and(Ti, Zr)(C, N) solid solutions. However, the first stage is still dominated by the reaction because of the low temperature. Therefore, TiC_0.5_N_0.5_ reacts with Si at this stage based on our previous research [10], as shown in Equation (2).

TiC_0.5_N_0.5_ (s) + Si (s) → SiC (s) + TiSi_2_ (s) + TiN (s) (2)

Second, the eutectic reaction of Si and TiSi_2_ forms the Ti-Si liquid phase as the temperature reaches 1330 °C [31]. Then, the non-reactive Si begins to melt at 1414 °C, and *α*-ZrSi transforms into *β*-ZrSi at 1460 °C [30]. *β*-ZrSi can dissolve in Ti-Si liquid to form the Zr–Ti–Si liquid phase. The carbon atoms in the ZrC matrix diffuse into the Zr–Ti–Si liquid phase as the temperature increases, forming SiC, ZrC_1−x_, and TiC_1−x_ [10]. In the process of heat and pressure preservation, the TiN generated in the first stage and the ZrC_1−x_ and TiC_1−x_ in the second stage gradually dissolve into the ZrC and TiC_0.5_N_0.5_ lattices and finally form (Zr, Ti)(C, N) and(Ti, Zr)(C, N) solid solutions (ss), as shown in Equation (3).

TiN (s) + ZrC_1−x_ (s) + TiC_1−*x*_ (s) + ZrC (s) + TiC_0.5_N_0.5_ (s) → (Zr,Ti)(C,N) (ss) + (Ti, Zr)(C, N) (ss) (3)

Compared with the other two specimens, the Si content in Z50T20S reached 20 mol%, causing the liquid phase of Zr-Ti-Si generated in the second stage to not entirely participate in the reaction, and the liquid phase residual transitioned to *β*-ZrSi during the cooling process. Therefore, *β*-ZrSi was found in the Z50T20S specimen.

### 3.2. Relative Densities

**Figure 4:**
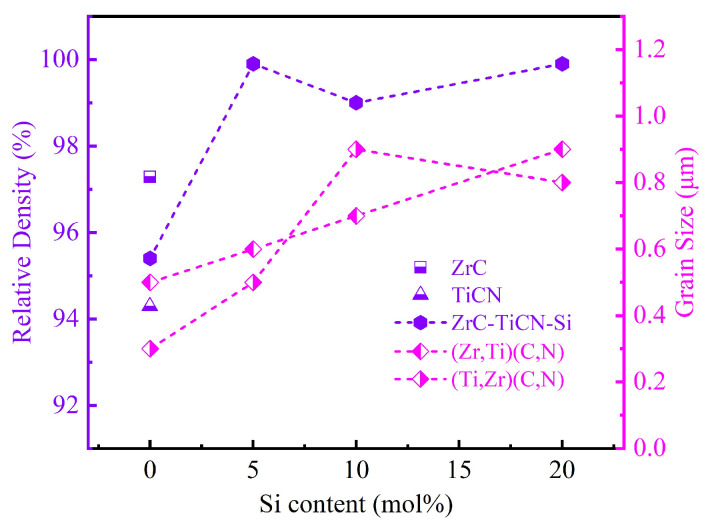
Relative densities and grain sizes of ceramics with various Si additions.

The curves of relative densities and grain sizes versus Si content for the prepared composites are shown in Figure 4. The relative density (RD) of pure ZrC sintered at 2100 °C was 97.3%. However, the RD decreased to 95.4% after 50 mol% TiC_0.5_N_0.5_ addition. As shown in Figure 3, the Z50T sample at the set sintering temperature was close to the “(Zr, Ti)(C, N) + SiC + N_2_ region” on the ZrC-TiC_0.5_N_0.5_-Si phase diagram. Therefore, the above phenomenon may be related to the appearance of N_2_. Similar phenomena have also occurred in other studies. Liang et al. [13] prepared the ceramic of the same composition with an RD of 96.1% by SPS at 2000 °C. They then measured nitrogen content under this composition, and the result showed that the loss of the nitrogen element reached 15 wt% after sintering. This demonstrates that TiC_0.5_N_0.5_ becomes unstable at higher temperatures and denitrifies to produce N_2._ Denitrification is worse when the amount of nitrogen and temperature are both high. The generated N_2_ will eventually form pores in the ceramic matrix at high sintering pressure. The minimum RD of the prepared TiC_0.5_N_0.5_ ceramics was 94.3%, consistent with the above analysis. As shown in Figure 4, despite the sintering temperatures being 300–500 °C lower for single-phase ceramics, the addition of Si significantly enhanced the RDs of (Zr, Ti)(C, N)-based composites to over 99%. According to the analysis of the reaction process, the presence of the liquid phase containing Ti, Zr, and Si enhanced the densification performance by promoting mass transfer. In addition, TiC_0.5_N_0.5_ is not sensitive to denitrification at 1500–1700 °C [12,18]. Meanwhile, the substitution of N for C easily tends to make (Zr, Ti)(C, N) solid solutions dense at a lower temperature (approximately 1700–1900 °C), and the (Ti, Zr)(C, N) solid solution formed when Zr substituted for Ti also has the same characteristic [12]. Thus, under the effect of the above factors, the Si-containing samples exhibited extremely high RDs, and the RD of Z50T20S even reached 99.9% at a sintering temperature of 1500 °C.

### 3.3. Microstructure

**Figure 5:**
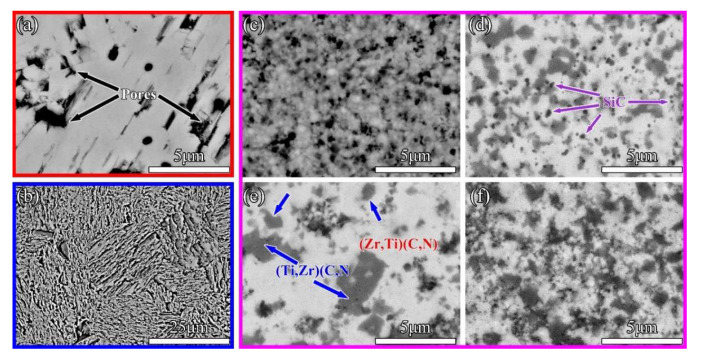
BSE micrographs of ceramics: (**a**) ZrC, (**b**) TiCN, (**c**) Z50T, (**d**) Z50T5S, (**e**) Z50T10S, and (**f**) Z50T20S.

**Figure 6:**
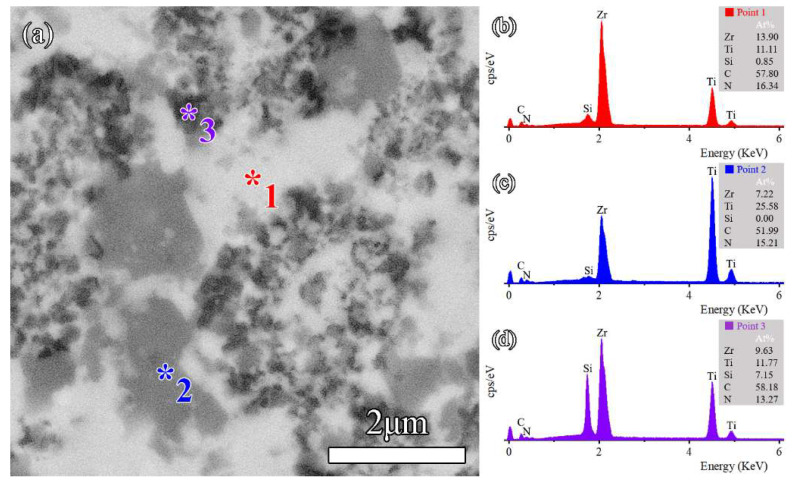
(**a**) BSE images of the Z50T10S and (**b**–**d**) EDS spectra taken from 1, 2, and 3 in (**a**).

SEM micrographs of sintered ceramics are shown in Figure 5. According to Figure 5a,b, it can be seen that the pure-phase ZrC and TiC_0.5_N_0.5_ sintered at 2100 °C had more surface porosity and poorer densification. Like pure ceramics, the Z50T sample exhibited high porosity, as shown in Figure 5c. With the addition of Si, the pores were significantly reduced, and the few apparent pores in these SEM images implied that these samples were close to being fully dense, as shown in Figure 5d–f. Meanwhile, dark and gray phases started to appear due to the introduction of TiCN and Si into the ZrC matrix. The EDS result of the Z50T10S sample is shown in Figure 6, and the combination of XRD and EDS analysis revealed that the bright, dark, and gray phases corresponded to (Zr, Ti)(C, N), (Ti, Zr)(C, N), and *β*-SiC, respectively. Likely attributable to the influence of solid solution factors, the contrast of (Ti, Zr)(C, N) and *β*-ZrSi was close (see the sequent HAADF images), so it could not be clearly distinguished on the BSE image of the Z50T20S sample.

Table 3 exhibits the content of phases in sintered ceramics calculated by the image analysis method. We previously reported that only (Zr, Ti)(C, N), and SiC existed in ceramic sintered at 1700 °C when the ratio of ZrC to Si was 90.25: 5 (mol%) in the ZrC-TiC_0.5_N_0.5_-Si system [10]. Therefore, it can be inferred that *β*-ZrSi does not exist in the Z50T5S ceramic, which has a phase composition of (Zr, Ti)(C, N)-31.2 vol% (Ti, Zr)(C, N)-4.2 vol% SiC. For the Z50T10S sample, the existence of *β*-ZrSi needs to be further analyzed using TEM. The contents of (Zr, Ti)(C, N) and (Ti, Zr)(C, N) solid solutions gradually decrease with increasing Si addition in (Zr, Ti)(C, N)-based composites. The average grain size of each phase in the sintered ceramics is shown in Table 3. The grain sizes of (Zr, Ti)(C, N), (Ti, Zr)(C, N), and SiC in Z50T5S were 0.6 ± 0.2, 0.5 ± 0.1, and 0.3 ± 0.1 μm, respectively, which were lower than those of ZrC-based ceramics sintered by conventional methods [32,33]. In particular, the grain sizes of (Zr, Ti)(C, N) and (Ti, Zr)(C, N) in the Z50T sample were 0.5 and 0.3 μm, respectively. Even though they had a low RD, these were the finest grain sizes in the sintered ceramics. The ZrC-TiCN ceramics prepared by Liang et al. showed a significant reduction in grain size since the Ti-rich solid solution was present [13], and the same phenomenon was found in ZrC-TiC ceramics [17,34]. Hence, it is thought that the presence of a Ti-rich solid solution in the ceramic matrix inhibits grain growth. According to Figure 4, the grain size of the two solid solution phases is positively correlated with the Si content. This is reasonable because the Si-containing liquid phase formed during the sintering process promotes mass transfer. Possibly due to the physical pinning effect of SiC, which was also reported in other ceramics with SiC or Si addition [35,36,37,38], the grain size of (Zr, Ti)(C, N) and (Ti, Zr)(C, N) did not grow excessively. The maximum grain size appeared in the Z50T20S sample at no more than 1 μm.

**Figure 7:**
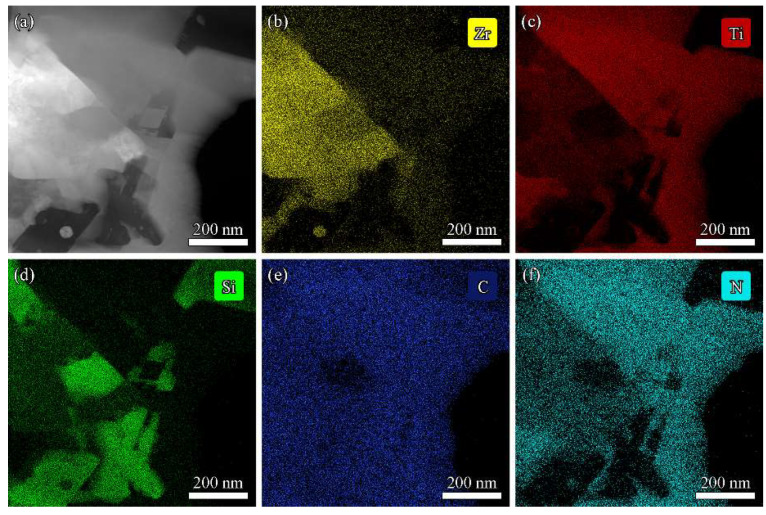
(**a**) HAADF image of Z50T10S. (**b**–**f**) STEM-EDS mapping of Zr, Ti, Si, C, and N, respectively.

**Figure 8:**
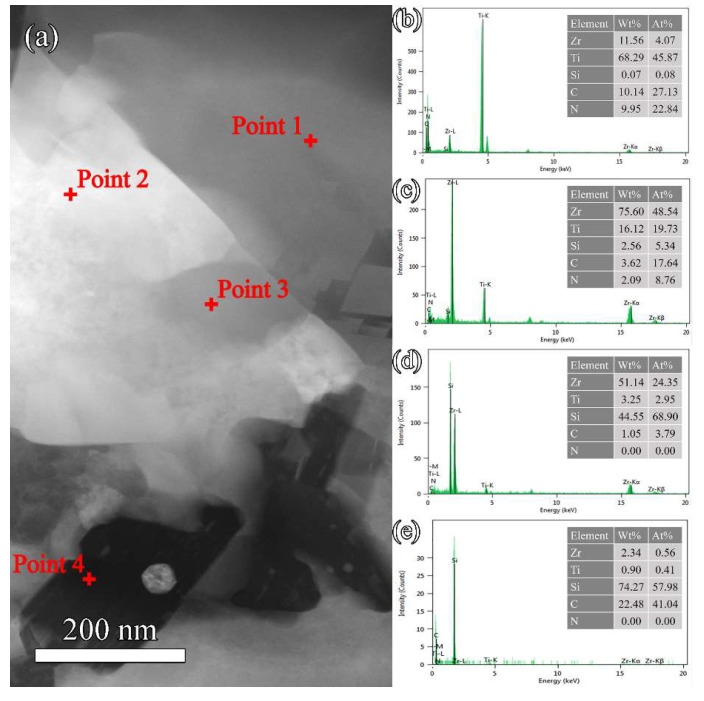
(**a**) HAADF image of the Z50T10S sample. (**b**–**e**) STEM-EDS spectra of points 1, 2, 3, and 4, respectively.

The Z50T10S was characterized by STEM to investigate the microstructure in detail. The HAADF image with element distribution maps and EDS spectra are shown in Figure 7 and Figure 8, respectively. The results showed that the phase with rich Si and C elements was SiC, which was aggregated due to the high content of Si in the system. At the grain boundary of (Zr, Ti)(C, N) and (Ti, Zr)(C, N), the gray nanoparticle with rich Si and poor C elements, almost embedded in the (Zr, Ti)(C, N) grain, corresponded to *β*-ZrSi. This indicated the existence of traces of *β*-ZrSi in the Z50T10S sample. According to the analysis of the reaction process in Section 3.1, it was formed by cooling the partially reacted liquid phase at the grain boundaries.

**Figure 9:**
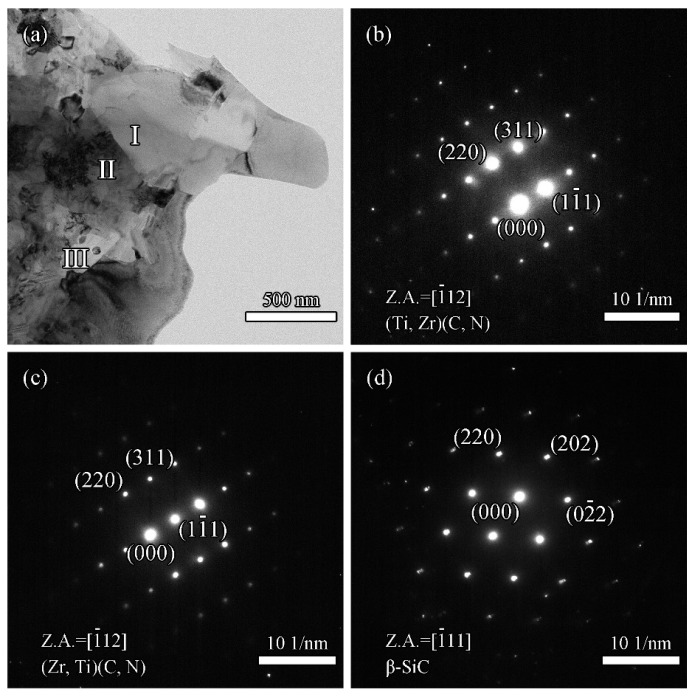
(**a**) TEM bright-field image of Z50T10S; (**b**–**d**) SAED patterns of regions I, II, and III, respectively.

From the EDS spectra in Figure 8b,c, it can be seen that the solubilities of Ti atoms in the ZrC lattice and Zr atoms in the TiC_0.5_N_0.5_ lattice were 28.90% and 8.15%, respectively. However, the solubility of Ti atoms in the *β*-ZrSi lattice is detected at 10.81%. It was revealed that the Ti atoms present in the *β*-ZrSi lattice could stabilize the high-temperature phase at room temperature. Figure 9 shows the selected area electron diffraction (SAED) patterns of Z50T10S. By indexing the diffraction spots, the bright phase, the larger gray phase, and the black phase were further determined as (Zr, Ti)(C, N), (Ti, Zr)(C, N), and *β*-SiC, respectively. SiC and ZrSi grains were surrounded by larger (Zr, Ti)(C, N) and (Ti, Zr)(C, N) grains, which formed a typical shape in-situ reaction. The grain sizes of (Zr, Ti)(C, N), (Ti, Zr)(C, N), and SiC (Figure 9a) were similar to the average value determined from the SEM image.

### 3.4. Mechanical Properties

The mechanical properties of monolithic ZrC, TiCN, and ZrC-SiC, and the ZrC-TiCN composites examined by the authors in [12,13,33,34,39,40,41,42], in addition to the results of this work, are summarized in Table 4. Compared to ZrC and TiCN [34,42], (Zr, Ti)(C, N) and (Ti, Zr)(C, N) ceramics [12,13] exhibited higher hardness due to the formation of complete solid solutions when sintered at high temperatures. The hardness of (Zr, Ti)(C, N) ceramics increases significantly with solid solubility. For the (Zr, Ti)(C, N) coatings with different non-metal/metal ratios deposited onto Ti_6_Al_4_V alloy and Si substrates by DC magnetron sputtering, it was reported that the hardness values of the films with thicknesses ranging from 1.8 to 2.1 µm reached 25–29 GPa [43]. This suggested that the hardness enhancement of the composite resulted from the intrinsically high hardness of (Zr, Ti)(C, N), which is presumed to be related to solid-solution-induced lattice distortion. XRD analysis showed that almost all TiC_0.5_N_0.5_ dissolved into the ZrC lattice in the Z50T, which resulted in a significant solid solution strengthening effect, improving hardness; therefore, a maximum hardness of 26.9 ± 0.7 GPa was attained. For the Z50T5S sintering at 1700 °C, a large amount of (Ti, Zr)(C, N) solid solution appeared in the matrix compared with the Z50T. Furthermore, the lower sintering temperature resulted in lower solid solubility. Z50T5S has a lower hardness than Z50T, with a hardness value of 24.5 ± 0.7 GPa. As the sintering temperature was further reduced, the hardness of Z50T10S and Z50T20S decreased. However, since the hardness of ZrSi was approximately 10 GPa [44], the hardness of Z50T20S decreased to 21.7 ± 0.7 GPa.

The flexural strength of Z50T was 297 ± 49 MPa lower than that of pure ZrC and (Zr, Ti)(C, N) [13] ceramics due to the poor RD. As Si was introduced into the ZrC-TiC_0.5_N_0.5_ system, the ceramics possessed a higher strength. The flexural strength of Z50T10S reached a value of 435 ± 28 MPa, which was higher than that of (Zr, Ti)(C, N) ceramics, as shown in Table 4. Besides high RD and solid solution strengthening, fracture toughness was also a significant factor of flaw tolerance according to the Irwin relationship, as shown by Equation (4):

(4) $$\sigma =\frac{K_{IC}}{Y\sqrt{a}}$$

where *σ*, *K_IC_*, *Y*, and *a* are the flexural strength, fracture toughness, geometric constant, and defect size of ceramic, respectively. Grain size is usually considered the characteristic critical defect size in almost completely dense ceramics. Thus, the higher flexural strength of ceramics with the Si addition may be correlated with the higher fracture toughness. Z50T5S exhibited a high RD and fine grain size, with a flexural strength of 508 ± 33 MPa, representing the maximum in prepared ceramics. Compared to ZrC-SiC ceramics [33,39,40,41], Z50TS contained 4.2 vol% SiC but had a significantly improved flexural strength that was generally higher than for other ceramics. This was not only due to the fine and dense microstructure but also the effective pinning impact of the grain boundaries caused by SiC generated in situ and distributed along the grain boundaries. In this study, however, the flexural strength of the other two ceramics containing Si was lower than that of Z50T5S, and the higher the Si content, the more severe the reduction in flexural strength. In (Zr, Ti)(C, N)-based ceramics, the mismatch in the coefficient of thermal expansion (CTE) between each phase led to the appearance of residual stresses during the cooling process. The CTEs of SiC, ZrC, TiC_0.5_N_0.5_, and ZrSi were 4.5 × 10^−6^, 6.8 × 10^−6^, 7.4 × 10^−6^, and 8.6 × 10^−6^ K^−1^, respectively [44,45]. Considering the relatively low concentration of solute atoms in (Zr, Ti)(C, N), (Ti, Zr)(C, N), and ZrSi, their CTEs can roughly refer to those of the above. According to Figure 8a, ZrSi was mainly distributed along the grain boundaries of (Zr, Ti)(C, N), and the CTE difference between ZrSi and (Zr, Ti)(C, N) was more significant than that of ZrSi and (Ti, Zr)(C, N). Therefore the residual stresses in ceramics were predominantly present at the ZrSi and (Zr, Ti)(C, N) grain boundaries. In the cooling process, the ZrSi particle was subjected to compressive stress, and the (Zr, Ti)(C, N) matrix was subjected to tensile stress, resulting in the interface being under tensile stress. Aggregating residual tensile stresses at grain boundaries induces microcracks, severely reducing the ceramic’s flexural strength. Apparently, the addition of Si is positively correlated with residual tensile stress-induced microcracks. The flexural strength of the Z50T20S ceramic was 330 ± 17 MPa, which was only slightly higher than that of pure ZrC.

**Figure 10:**
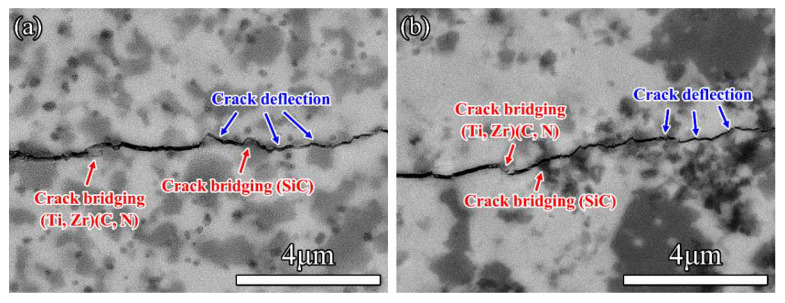
Images of crack propagation of (**a**) Z50T5S and (**b**) Z50T10S.

The content of (Zr, Ti)(C, N) solid solution in Z50T ceramics is much higher than that of (Ti, Zr)(C, N) solid solution, so (Ti, Zr)(C, N) can be regarded as the second phase, similar to SiC and ZrSi. Compared with pure ZrC and TiCN ceramics, the fracture toughness of Z50T was improved to 3.3 ± 0.1 MPa·m^1/2^. With the Si addition increased, the fracture toughness of composites (20 mol% Si addition) increased to 4.4 ± 0.2 MPa·m^1/2^, caused by a significant rise in the fraction of the dispersed second phase in the matrix [46]. As shown in crack propagation morphologies (Figure 10), Ti-rich (Ti, Zr)(C, N) particles can cause crack deflection and bridging, in which toughening is achieved by consuming the energy for the primary crack propagation and reducing the stress accumulation at the crack tip. The CTE values of ZrC and SiC indicate that the SiC in the ceramic is subjected to residual tensile stress at room temperature, and tangential tensile stress around embedded SiC particles is always greater than intergranular SiC [47]. Under tensile stress, SiC particles can pull the propagating crack, resulting in particle bridging and toughening. Moreover, microcracks induced by residual stress at the grain boundaries between (Zr, Ti)(C, N) and ZrSi play an important role in enhancing the fracture toughness of ceramic composites. As the matrix is subjected to tensile stress, a plastic zone is formed at the tip of the primary crack, and the microcracks in this zone propagate to create new cracks and consume energy. Simultaneously, in encountering larger microcracks with the main crack, the propagating path of the main crack is deflected and branched, resulting in a longer expansion path. This absorbs more energy, thus causing toughening. In conclusion, crack deflection and particle bridging are thought to be the dominant toughening mechanisms for (Zr, Ti)(C, N)-based ceramics.

## 4. Conclusions

We produced almost completely dense (Zr, Ti)(C, N)-based nanocomposites via reactive hot pressing at 1500–1700 °C using ZrC, TiC_0.5_N_0.5_, and Si powders as the raw materials. The addition of Si significantly reduced the sintering temperature due to the liquid phase formation during sintering. At the low sintering temperature, both (Zr, Ti)(C, N) and (Ti, Zr)(C, N) solid solutions appeared in the composites, and the mechanical properties were enhanced by adjusting the ratio of ZrC to TiC_0.5_N_0.5_. The liquid phase generated and the (Zr, Ti)(C, N) and (Ti, Zr)(C, N) solid solutions were conducive to facilitating the mass transfer and promoting densification. During the sintering process, all of the Si that was added in a 5 mol% amount turned into SiC. With the increased Si addition, the residual ZrSi phase appeared, reducing the flexural strength and hardness of composites while improving their toughness. (Ti, Zr)(C, N) and SiC effectively inhibited the growth of matrix grains. The (Zr, Ti)(C, N)-based composite with 47.5 mol% ZrC, 47.5 mol% TiC_0.5_N_0.5_, and 5 mol% Si possessed excellent comprehensive mechanical properties (flexural strength of 508 ± 33 MPa, Vickers hardness of 24.5 ± 0.7 GPa, and fracture toughness of 3.8 ± 0.1 MPa·m^1/2^). The higher flexural strength and hardness were mainly attributed to low porosity, refined grains, solid solution strengthening, and second-phase strengthening; meanwhile, the crack deflection and bridging caused by the (Ti, Zr)(C, N) and SiC particles resulted in higher toughness.

## Figures and Tables

**Table 1:** Designed compositions and sintering parameters of composites.

Code	Compositions of Composites				Sintering Parameters
	ZrC:TiCN	ZrC (mol%)	TiCN (mol%)	Si (mol%)	
ZrC	100:0	100	-	-	2100 °C/30 MPa/1 h
TiCN	0:100	-	100	-	2100 °C/30 MPa/1 h
Z50T	50:50	50	50	-	2000 °C/30 MPa/1 h
Z50T5S	50:50	47.5	47.5	5	1700 °C/30 MPa/1 h
Z50T10S	50:50	45	45	10	1500 °C/30 MPa/1 h
Z50T20S	50:50	40	40	20	1500 °C/30 MPa/1 h

**Table 2:** Diffraction peak positions and lattice parameters of sintered ceramics.

Composition	2θ (°) of (220)		2θ (°) of ZrSi (002)	Lattice Parameters (nm)	
	ZrC/ (Zr,Ti)(C,N)	TiCN/ (Ti,Zr)(C,N)		ZrC/ (Zr,Ti)(C,N)	TiCN/ (Ti,Zr)(C,N)
ZrC (PDF # 00-035-0784)	55.3	-	-	0.4693	-
TiC_0.51_N_0.49_ (PDF # 04-006-0748)	-	61.2	-	-	0.4278
*β*-ZrSi (PDF # 04-004-7165)	-	-	48.5	-	-
ZrC	55.3	-	-	0.4696	-
TiCN	-	61.2	-	-	0.4275
Z50T	57.5	59.1	-	0.4525	0.4418
Z50T5S	57.0	59.9	-	0.4561	0.4356
Z50T10S	56.7	60.2	-	0.4587	0.4350
Z50T20S	56.7	60.2	49.5	0.4589	0.4346

**Table 3:** Grain sizes and phase contents by image analysis of ceramics.

Sample	Relative Density (%)	Grain Size (μm)			Phase Content by Image Analysis (vol%)			
		ZrC/ (Zr, Ti)(C, N)	TiCN/ (Ti, Zr)(C, N)	SiC	ZrC/ (Zr, Ti)(C, N)	TiCN/ (Ti, Zr)(C, N)	SiC	ZrSi
ZrC	97.3	1.3 ± 0.2	-	-	100	-	-	-
TiCN	94.3	-	24.2 ± 5.7	-	-	100	-	-
Z50T	95.4	0.5 ± 0.1	0.3 ± 0.1	-	88.1	11.9	-	-
Z50T5S	99.9	0.6 ± 0.2	0.5 ± 0.1	0.3 ± 0.1	64.6	31.2	4.2	-
Z50T10S	99.0	0.7 ± 0.2	0.9 ± 0.3	0.4 ± 0.1	57.1	-	9.8	trace
Z50T20S	99.9	0.9 ± 0.2	0.8 ± 0.1	0.3 ± 0.1	54.7	-	12.5	-

**Table 4:** The mechanical properties of ceramics of this paper and previous reports [12,13,33,34,39,40,41,42].

Sample	Processing Parameters	Relative Density (%)	Grain Size (μm)			Flexural Strength (MPa)	Vickers Hardness (GPa)	Fracture Toughness (MPa·m^1/2^)	Ref.
			ZrC/ (Zr,Ti)(C,N)	TiCN/ (Ti,Zr)(C,N)	SiC				
ZrC	HP/2200 °C/1 h/30 MPa	98.2	~40	-	-	210 ± 19	16.4 ± 1.3	3.1 ± 0.7	[34]
ZrC-10 vol% SiC	HP/1900 °C/1 h/32 MPa	98.2	1.4 ± 0.6	-	0.5 ± 0.2	~405	19.7 ± 0.6	2.9 ± 0.1	[33]
ZrC-20 vol% SiC	HP/1900 °C/1 h/30 MPa	95	~10	-	~4	~450	~19.6	~3.9	[39]
ZrC-20 vol% SiC	HP/1900 °C/1 h/32 MPa	98.3	1.0 ± 0.5	-	0.5 ± 0.2	~427	~21.2	2.9 ± 0.1	[33]
ZrC-30 vol% SiC	HP/1900 °C/1 h/32 MPa	99.3	1.5 ± 0.6	-	0.8 ± 0.5	-	23.0 ± 0.5	2.6 ± 0.2	[40]
ZrC-30 vol% SiC	SPS/1800 °C/5 min/45 MPa	96.1	<1.0	-	~0.5	523 ± 20	18.8 ± 1.2	4.0 ± 0.3	[41]
TiCN	SPS/1800 °C/5 min/75 MPa	98.6	-	3.4 ± 1.5	-	-	21.8 ± 1.3	2.5 ± 0.3	[42]
ZrC-90 mol% TiCN	SPS/2000 °C/5 min/50 MPa	98.0	-	-	-	-	20.3	2.7	[12]
ZrC-80 mol% TiCN		99.0	-	-	-	-	22.2	2.0	
ZrC-5 mol% TiCN	SPS/2000 °C/10 min/40 MPa	97.4	1.1 ± 0.2	-	-	378 ± 18	16.4 ± 2.0	2.3 ± 0.3	[13]
ZrC-20 mol% TiCN		97.8	1.3 ± 0.4	-	-	368 ± 43	20.0 ± 0.8	2.5 ± 0.2	
ZrC-50 mol% TiCN		96.1	0.3 ± 0.1	0.3 ± 0.1	-	305 ± 41	22.5 ± 1.0	3.4 ± 0.3	
ZrC	HP/2100 °C/30 MPa/1 h	97.3	1.3 ± 0.2	-	-	312 ± 35	16.7 ± 1.0	2.8 ± 0.3	This work
TiCN	HP/2100 °C/30 MPa/1 h	94.3	-	24.2 ± 5.7	-	280 ± 31	21.0 ± 1.8	2.6 ± 0.5	
Z50T	HP/2000 °C/30 MPa/1 h	95.4	0.5 ± 0.1	0.3 ± 0.1	-	297 ± 49	26.9 ± 0.7	3.3 ± 0.1	
Z50T5S	HP/1700 °C/30 MPa/1 h	99.9	0.6 ± 0.2	0.5 ± 0.1	0.3 ± 0.1	508 ± 33	24.5 ± 0.7	3.8 ± 0.1	
Z50T10S	HP/1500 °C/30 MPa/1 h	99.0	0.7 ± 0.2	0.9 ± 0.3	0.4 ± 0.1	435 ± 28	22.4 ± 0.5	4.0 ± 0.3	
Z50T20S	HP/1500 °C/30 MPa/1 h	99.9	0.9 ± 0.2	0.8 ± 0.1	0.3 ± 0.1	330 ± 17	21.7 ± 0.7	4.4 ± 0.2	

## Data Availability

Not applicable.
